# Subject Specific Optimisation of the Stiffness of Footwear Material for Maximum Plantar Pressure Reduction

**DOI:** 10.1007/s10439-017-1826-4

**Published:** 2017-05-09

**Authors:** Panagiotis E. Chatzistergos, Roozbeh Naemi, Aoife Healy, Peter Gerth, Nachiappan Chockalingam

**Affiliations:** 1 School of Life Sciences and Education, Department of Sport and Exercise, Staffordshire University, Leek Road, Stoke-on-Trent, ST4 2DF UK; 2 Magdeburg-Stendal University of Applied Sciences, Magdeburg, Germany

**Keywords:** Diabetic foot, *In vivo* testing, Polyurethane foam, Shoe, Insole, Orthotic devices, Pressure measurement, Biomechanics, Clinical management

## Abstract

Current selection of cushioning materials for therapeutic footwear and orthoses is based on empirical and anecdotal evidence. The aim of this investigation is to assess the biomechanical properties of carefully selected cushioning materials and to establish the basis for patient-specific material optimisation. For this purpose, bespoke cushioning materials with qualitatively similar mechanical behaviour but different stiffness were produced. Healthy volunteers were asked to stand and walk on materials with varying stiffness and their capacity for pressure reduction was assessed. Mechanical testing using a surrogate heel model was employed to investigate the effect of loading on optimum stiffness. Results indicated that optimising the stiffness of cushioning materials improved pressure reduction during standing and walking by at least 16 and 19% respectively. Moreover, the optimum stiffness was strongly correlated to body mass (BM) and body mass index (BMI), with stiffer materials needed in the case of people with higher BM or BMI. Mechanical testing confirmed that optimum stiffness increases with the magnitude of compressive loading. For the first time, this study provides quantitative data to support the importance of stiffness optimisation in cushioning materials and sets the basis for methods to inform optimum material selection in the clinic.

**Electronic supplementary material:**

The online version of this article (doi:10.1007/s10439-017-1826-4) contains supplementary material, which is available to authorized users.

## Introduction

The redistribution of plantar loads to reduce plantar pressure is one of the main therapeutic objectives for the management of the diabetic foot syndrome and therapeutic footwear/orthoses play an important role in facilitating this objective.^13^ Considering their role, it is no surprise that the effectiveness of therapeutic footwear and/or orthoses is strongly dependent on the mechanical characteristics of the materials that are used to offer cushioning, with stiffness being among the most critical characteristics.^6^,^9^,^12^,^15^,^20^,^21^

Even though careful selection of stiffness of cushioning materials is highlighted as an imperative factor in order to achieve maximum pressure reduction,^6^,^9^,^20^ currently no set method exists to inform this selection process.^12^ Indeed material selection in the clinic is solely based on empirical or anecdotal evidence^15^ and no clear guidelines are available to enable healthcare professionals, working in the area of diabetic foot care, to decide whether a specific material is adequately “soft” or “stiff” on a patient specific basis.

In this context, a recent numerical analysis aimed to shed light on the optimisation of stiffness of cushioning materials to minimise plantar pressure and to assess the effect of various patient specific parameters on optimum stiffness.^8^ More specifically, the parameters that were investigated in this study^8^ were: (a) plantar soft tissue stiffness (b) plantar soft tissue thickness and (c) the magnitude of plantar loads. This analysis revealed that the stiffness of the insole that can minimise pressure (i.e., optimum insole stiffness) is strongly affected by the magnitude of loading, with stiffer insoles minimising plantar pressure in the case of higher loads. On the contrary, optimum insole stiffness appeared not to be affected by the stiffness or by the thickness of plantar soft tissue. These findings highlighted patient specific loading as a possible critical factor to inform the selection of cushioning material stiffness.^8^

Building on this numerical work the present study aims to investigate *in vivo* the effect of cushioning material stiffness on footwear’s capacity to reduce planar pressure and to quantitatively assess the importance of systematic material selection. Moreover, the possibility of optimising stiffness on a patient specific basis will also be explored by investigating the associations between optimum stiffness and anthropometric parameters that are routinely measured in the clinic.

## Materials and Methods

### Insole Materials and Mechanical Testing

A range of bespoke polyurethane (BPU) foams with similar qualitative mechanical characteristics but with different stiffness was manufactured using commercially available chemical compounds (AstiS, AstiTech^®^ 150 and AstiTech^®^ 300, BEIL GmbH, Moderne Orthopädie Kunststoffe, Germany). More specifically, ten different compositions were used for manufacturing ten different BPU foam materials in the form of rectilinear sheets with dimensions 400 mm × 400 mm × 10 mm. A detailed description of the manufacturing process is presented in Supplementary Material (S1).

**Figure 1:**
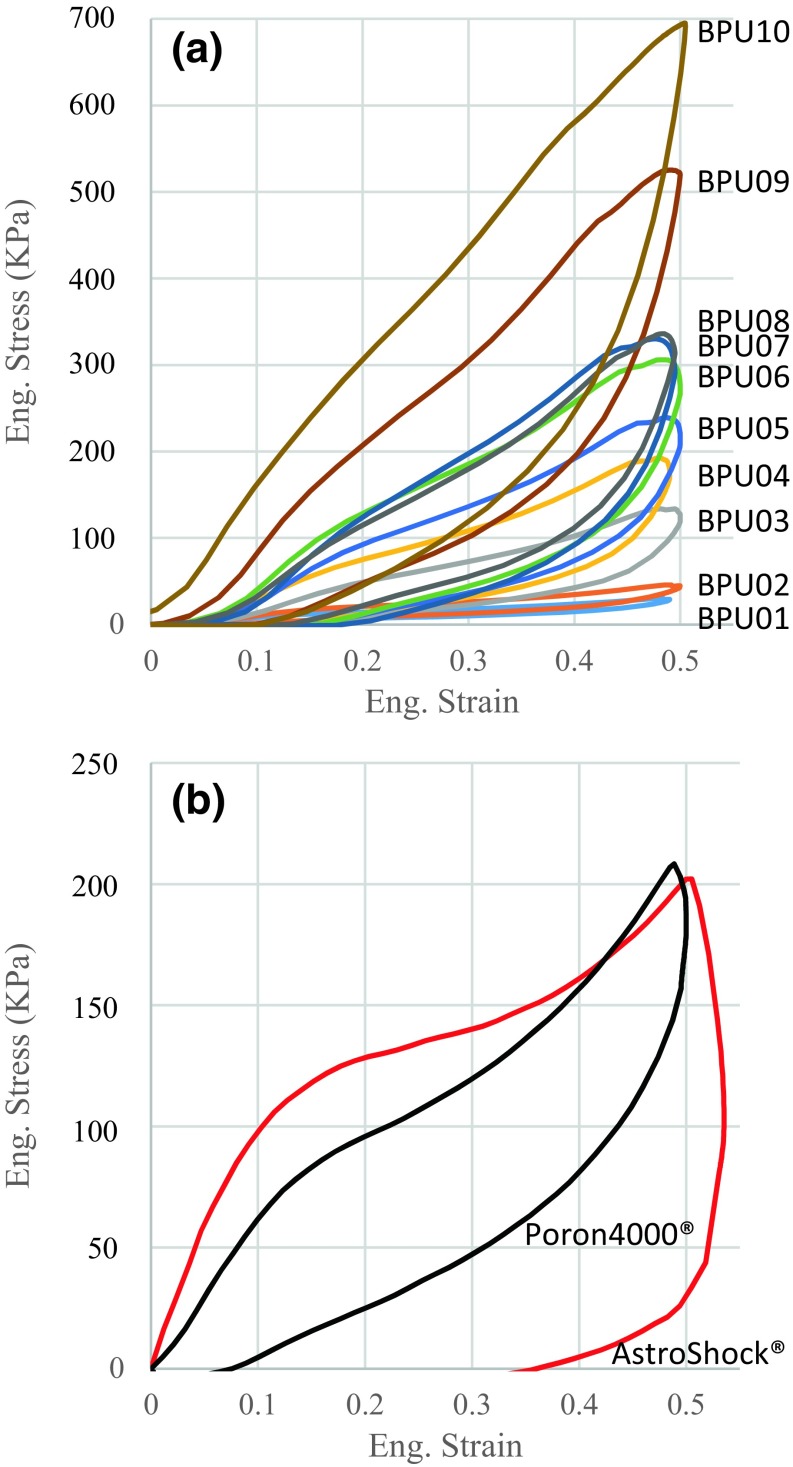
Stress (kPa)/strain (unitless) graphs for the bespoke polyurethane foam materials (BPU01-10) (a) and the two commercially ones (Poron^®^4000, AstroShock^®^) that were used as reference (b).

The mechanical characteristics of the produced material were assessed in a series of mechanical tests and compared to two commercial materials that are commonly used in therapeutic footwear, namely: AstroShock^®^ and Poron^®^4000. More specifically cylindrical samples were cut using a punch with diameter of 30 mm and subjected to quasi-static and dynamic compression at loading rates of 0.008 and 30 mm/s respectively.^10^,^17^ Each test was repeated five times and the relative stiffness was quantified using the average value of compressive stress for 50% dynamic compression (Fig. 1). Stress for 50% compression was used to arrange the BPU materials from soft to stiff and inform their labelling with BPU01 being the softest and BPU10 the stiffest one. Shore A hardness was also measured using an analogue durometer (Zwick 3114, Zwick GmbH &Co).

**Figure 2:**
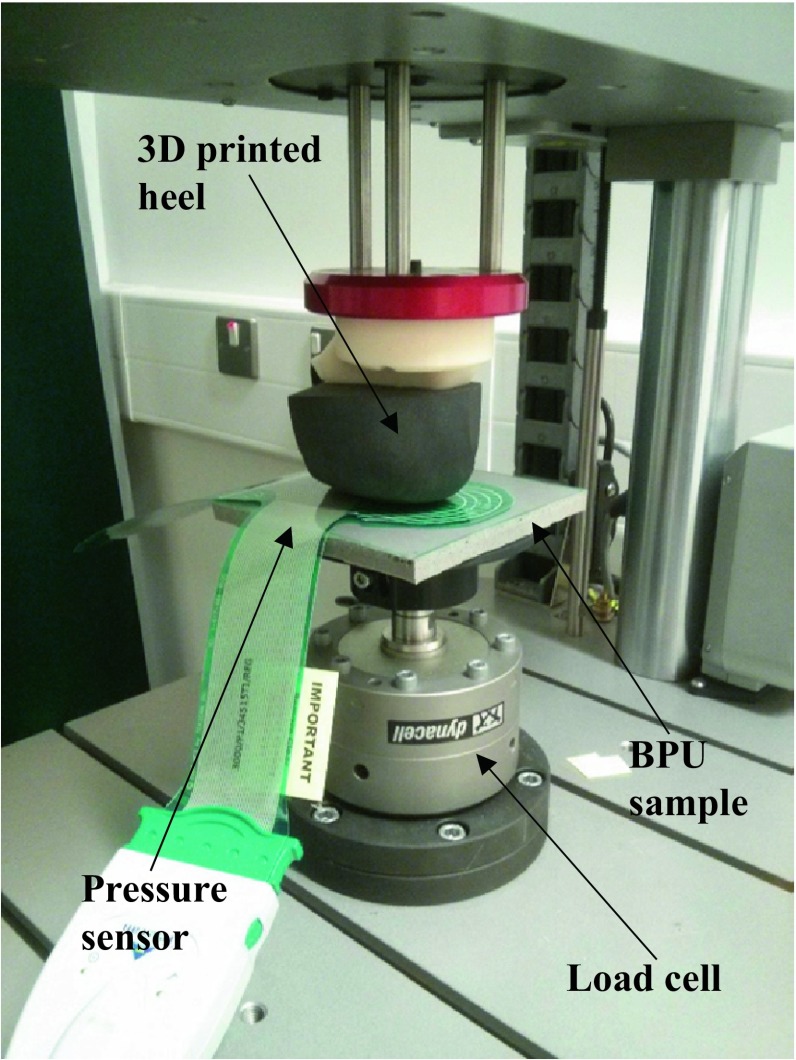
Testing set-up for investigating the effect of loading on the ability of cushioning materials to uniformly distribute plantar loads using a 3D printed heel model.

The effect of loading on the ability of BPU materials to uniformly distribute plantar loads was first assessed in a series of mechanical tests using an anatomically accurate physical model of a heel (Fig. 2). For the manufacturing of the heel model, the foot of one healthy volunteer was imaged using a 1.5 T MRI scanner and a series of coronal T1 weighted 3D Fast Field Echo (FFE) images were recorded with in-plane and out of plane resolution of 0.23 and 1.00 mm respectively. The areas of the calcaneus and bulk soft tissue were segmented on each image of the heel and their 3D shapes were reconstructed using ScanIP (Simpleware). A 3D printer was used to manufacture the heel model as a single part using rigid material for the volume of the calcaneus and a deformable rubber-like material with shore A hardness equal to 20 ± 1 as bulk soft tissue.

Rectangular samples with dimension of 130 mm × 130 mm were cut from the BPU material sheets and compressed between a rigid plate and the heel model (Fig. 2). Loading was applied using a 3 kN INSTRON ElectroPuls™ E3000 load frame while peak pressure between the heel model and the cushioning material was measured using an F-scan sensor (F-scan^®^, Tekscan, Boston, MA, US). F-scan sensors are ultra-thin pressure sensors (0.15 mm) with 960 sensing locations over their entire surface providing high spatial resolution (four sensing locations per cm^2^).

After preconditioning, peak pressure and net compressive force were recorded for three load/unload cycles. In order to fully understand the relationship between loading magnitude and peak pressure, maximum compressive displacement of 12 mm from initial contact was imposed to the complete bottoming-out of the 10 mm thick insole materials. Based on preliminary tests, a safety force limit of 600 N also had to be defined to ensure the structural integrity of the 3D printed components. After completing the tests, peak pressure during loading was plotted over net force for all load cycles. For each material a high order polynomial was fitted to the data from all three load cycles to represent the relationship between peak pressure and net compressive force.

### *In Vivo* Testing

Ten healthy volunteers were recruited for the purpose of this study with average (±STDEV) age of 39 (±11) years. As it can be seen in Table 1 the recruited sample was very diverse in terms of their body mass (BM), height and shoe size. Ethical approval was sought and granted by the University’s Ethics Committee and all participants provided full informed consent.

**Table 1:** Characteristics of the participants of this study.

Volunteer	Sex (M/F)	Age (years)	BM (kg)	Height (cm)	BMI	Shoe size (EU)
#1	F	29	54	155	23	38
#2	M	29	54	175	18	42
#3	M	48	67	168	24	43
#4	M	46	124	177	40	46
#5	M	23	75	169	26	43
#6	M	62	121	182	37	46
#7	F	33	70	164	26	40
#8	M	39	80	178	25	43
#9	M	37	83	189	23	46
#10	F	41	65	175	21	40
Average		39	79	173	26	43
STDEV		11	25	10	7	3

The participants firstly stood barefoot on each of the BPU materials while plantar pressure was measured using F-scan sensors, which were calibrated to the manufacturer’s guidelines for each participant. For these measurements, the material BPU10 which is practically rigid was used as reference to assess peak pressure reduction for the remaining nine BPU materials. Materials were tested in random order and each test was repeated three times. For each trial plantar pressure distribution was recorded at 100 Hz while the participants stood still for 15 s. After the end of the tests the peak pressure of the entire foot (independent of location) was averaged for the three trials for each material tested and the pressure reduction relative to the rigid BPU10 was assessed for all nine remaining BPU materials. These calculations were performed for left and right foot separately but the overall performance of each material was ultimately assessed for the most heavily loaded foot, as defined based on the measured average peak pressure for the reference BPU material (i.e., BPU10). For simplicity, average peak pressure for standing will be, from this point on, referred to simply as static peak pressure.

The participants then wore plimsolls (i.e., minimalistic athletic shoes with a canvas upper and rubber sole) fitted with insoles that were cut from each of the BPU materials and in-shoe pressure distribution was measured during walking at a self-selected speed. More specifically walking speed was measured using timing gates (TC-PhotoGate A, Brower timing systems) placed at appropriate places to cover a distance of 5.8 m. Before the start of any pressure measurement, all participants were asked to walk the length between the timing gates five times. The time of the last three walking trials was averaged and used to assess preferred walking speed. Plantar pressure was then recorded using F-scan sensors sampling at 100 Hz. For each condition (i.e., BPU material) three walking trails were performed with at least four mid-gait steps each. In total, plantar pressure was recorded for at least twelve mid-gait steps per foot.^3^ Trials where walking speed was more than 10% lower or higher than the preferred walking speed were rejected and repeated. Pressure reduction was assessed relative to material BPU10 for each foot. The overall performance of each material was ultimately assessed for the heaviest loaded foot, namely the foot with the highest reference peak pressure.

Following testing, the average maximum peak pressure for each material was calculated. To calculate this value, firstly, the peak pressure for each one of the three trials (per condition) was calculated. Peak pressure is defined as the highest pressure in an area of 2 × 2 sensor-cells and it corresponds to the maximum pressure developed on a discrete point of the entire surface of the foot. Secondly, the mean peak pressure graph for each trial was then produced, where the measured peak pressures for different instances of gait over all mid-gait steps of each trial were averaged. The mean peak pressure graph is generated using a standardised process of F-scan software. Finally, average maximum peak pressure for each material was calculated by averaging the respective maximum peak pressures over the three trials. Pressure reduction was assessed relative to material BPU10 for each foot. The overall performance of each material was ultimately assessed for the heaviest loaded foot, namely the foot with the highest reference average maximum peak pressure. For simplicity, average maximum peak pressure for walking will be, from this point on, referred to simply as dynamic peak pressure

The aforementioned dynamic tests were performed a few days after the initial static ones and after a preliminary analysis of results. This preliminary analysis of the results for quiet standing enabled the exclusion of materials that were clearly too stiff for specific individuals, thus reducing the total duration of testing.

One way repeated measures ANOVA (statistical significance level = 0.05) with Bonferroni confidence interval adjustment was performed to investigate the importance of selecting insole material with optimum stiffness. For this purpose the pressure reduction achieved for the optimum material (i.e., maximum pressure reduction) was compared against the respective reduction achieved by the next softer and next stiffer material. Preliminary analyses were performed to ensure the assumptions of normality, linearity and homoscedasticity were not violated. Moreover the correlation between optimum insole stiffness, reference static or dynamic peak pressure (i.e., peak pressure for material BPU10) and various anthropometric parameters such as body mass (BM), height, body mass index (BMI) and shoe size was investigated using Pearson correlation analysis with bootstrapping (Statistical significance level = 0.05). In the case of walking the correlation between optimum stiffness and preferred walking speed was also investigated. Bootstrapping is a non-parametric resampling procedure that produces a distribution-free estimate of confidence intervals.^4^,^11^ Preliminary analyses were performed to ensure the assumptions of linearity and homoscedasticity were not violated. All statistical analyses were performed using IBM^®^ SPSS^®^v.21.

## Results

### Insole Materials and Mechanical Testing

Compressive tests using cylindrical samples showed that, in qualitative terms, the BPU materials appear to exhibit very similar stress/strain behaviour (Fig. 1). Comparison with the two commercial materials indicates that the range of stiffness of the BPU materials is also relevant to what is currently used in therapeutic footwear. As it can be seen in Table 2 the Shore A hardness of Poron^®^ 4000 and Astroshock^®^ is similar to BPU03 and their relative stiffness (i.e., stress at 50% dynamic compression) was similar to BPU04.

**Table 2:** Comparative results from mechanical testing for all bespoke materials (i.e., BPU01-10) as well as for two commercial ones (i.e., AstroShoch^®^ and Poron4000^®^) used as reference.

Material	Stress for 50% compression (kPa)		Hysteresis ratio	Shore A
	Quasi-static	Dynamic		
AstroShock^®^	82 ± 1	201 ± 20	0.881 ± 0.013	10 ± 1
Poron 4000^®^	141 ± 2	207 ± 12	0.526 ± 0.010	11 ± 1
BPU01	25 ± 1	36 ± 2	0.388 ± 0.010	2 ± 1
BPU02	36 ± 2	51 ± 2	0.448 ± 0.007	3 ± 2
BPU03	99 ± 2	155 ± 4	0.616 ± 0.007	10 ± 1
BPU04	149 ± 4	227 ± 4	0.624 ± 0.006	14 ± 1
BPU05	185 ± 3	276 ± 3	0.644 ± 0.003	18 ± 2
BPU06	237 ± 3	350 ± 5	0.666 ± 0.008	20 ± 1
BPU07	246 ± 9	367 ± 15	0.704 ± 0.012	24 ± 1
BPU08	281 ± 6	382 ± 7	0.589 ± 0.006	23 ± 1
BPU09	480 ± 10	598 ± 9	0.584 ± 0.013	37 ± 1
BPU10	611 ± 34	761 ± 32	0.630 ± 0.006	40 ± 1

Stress for 50% compression was used as a measure of relative stiffness

In the case of compression using a heel model, peak pressure and net force were recorded until the maximum imposed compressive displacement (i.e., 12 mm) only in five samples (materials BPU01-05). Curve fitting using 5th order polynomials produced very good fit with average *R* ^2^ (±STDEV) value of 0.996 (±0.001). For the remaining five out of ten materials, the tests were not successfully completed (materials BPU06-10) because the safety force limit was tripped.

**Figure 3:**
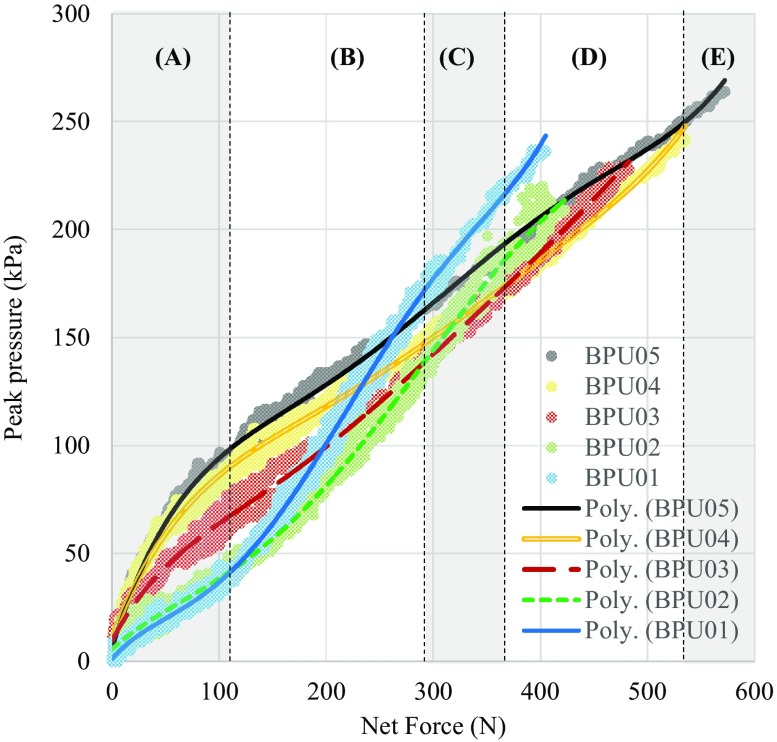
The relationship between externally applied net force and resulted peak pressure between the 3D printed heel model and different BPU materials. Results for three loading cycles are presented in each case. The 5th order polynomials that were fitted to the data are also shown.

As shown in Fig. 3, after an initial almost linear increase of peak pressure the slope of the graph of peak pressure over net force gradually drops only to rapidly increase again as the foam material starts to bottom-out. Comparing peak pressure for the same value of net force between materials indicates that the material that minimises peak pressure changes with the magnitude of force (Fig. 3). Using the equations of the fitted 5th order polynomials the graph in Fig. 3 can be divided into areas A–E where pressure is minimised by materials BPU01-05, respectively. For example in the case of net force equal to 100 N the material achieving optimum distribution of loads is BPU01 with peak pressure of just 37 kPa. At the same time the peak pressure for the same force for material BPU04 is 86 kPa. However when net force is increased to 400 N, BPU01 appears to lose its capacity to uniformly distribute loads and its peak pressure increases to 239 kPa. This time optimum cushioning is achieved by BPU04 with peak pressure equal to 186 kPa (Fig. 3). Based on this it can be concluded that stiffer materials are needed in the case of higher loads in order to minimise peak pressure.

### *In Vivo* Testing

#### Standing

Pressure measurements for quiet standing showed that static peak pressure for left and right foot were, in many cases, significantly different. Indeed, in the case of BPU10 the average absolute difference between left and right was 8% with eight out of ten participants putting more load on their left foot while standing. Considering the aforementioned differences in loading and the results from mechanical testing it is no surprise that in seven out of ten cases the material that minimised static peak pressure was different for the left and right foot (Table 3). However, focusing on the most heavily loaded foot enables identifying one optimum material for each participant. From this point on the material achieving maximum pressure reduction for the most heavily loaded foot will be referred to simply as the optimum material.

**Table 3:** Average (±STDEV) peak pressure for quiet standing (static peak pressure) on different BPU materials for all participants.

Participant	Foot	Static peak pressure (kPa)									
		BPU01	BPU02	BPU03	BPU04	BPU05	BPU06	BPU07	BPU08	BPU09	BPU10 (reference)
#1	Left	65 ± 11	*54* ± *4*	75 ± 13	80 ± 6	87 ± 14	90 ± 10	82 ± 9	96 ± 23	78 ± 4	101 ± 4
	Right^a^	52 ± 3	***48*** ± ***10***	76 ± 7	78 ± 6	83 ± 14	93 ± 11	78 ± 10	89 ± 7	91 ± 13	104 ± 12
#2	Left^a^	***92*** ± ***9***	113 ± 34	103 ± 36	156 ± 10	138 ± 12	143 ± 21	154 ± 39	165 ± 27	177 ± 42	186 ± 13
	Right	89 ± 31	*85* ± *31*	96 ± 26	125 ± 12	127 ± 19	116 ± 27	133 ± 30	146 ± 4	151 ± 26	145 ± 4
#3	Left^a^	97 ± 19	***84*** ± ***23***	113 ± 19	116 ± 10	144 ± 17	150 ± 20	149 ± 23	130 ± 30	148 ± 22	176 ± 35
	Right	*59* ± *6*	63 ± 11	81 ± 4	89 ± 9	94 ± 5	115 ± 1	98 ± 5	106 ± 8	101 ± 5	110 ± 9
#4	Left^a^	145 ± 19	143 ± 18	***127*** ± ***24***	149 ± 18	162 ± 24	158 ± 20	175 ± 22	165 ± 11	172 ± 23	189 ± 32
	Right	*76* ± *5*	78 ± 18	83 ± 9	103 ± 18	107 ± 8	94 ± 15	120 ± 11	109 ± 15	103 ± 21	110 ± 33
#5	Left^a^	103 ± 12	94 ± 12	***86*** ± ***5***	92 ± 4	101 ± 7	105 ± 10	107 ± 10	95 ± 8	106 ± 7	115 ± 15
	Right	60 ± 10	*55* ± *5*	76 ± 3	78 ± 10	85 ± 12	84 ± 8	97 ± 11	90 ± 2	88 ± 13	90 ± 19
#6	Left^a^	152 ± 14	130 ± 11	***126*** ± ***18***	127 ± 5	155 ± 17	173 ± 2	153 ± 22	157 ± 7	174 ± 9	165 ± 13
	Right	*77* ± *5*	77 ± 14	88 ± 4	98 ± 2	105 ± 4	120 ± 19	116 ± 7	122 ± 13	127 ± 10	139 ± 22
#7	Left	*71* ± *6*	72 ± 6	97 ± 2	95 ± 7	104 ± 7	105 ± 7	106 ± 14	105 ± 16	108 ± 14	122 ± 4
	Right^a^	91 ± 6	***80*** ± ***2***	107 ± 2	123 ± 10	148 ± 9	161 ± 6	150 ± 2	147 ± 16	175 ± 10	229 ± 15
#8	Left^a^	121 ± 13	***118*** ± ***15***	124 ± 9	131 ± 17	140 ± 6	134 ± 5	155 ± 4	139 ± 18	164 ± 16	163 ± 4
	Right	108 ± 21	*105* ± *10*	131 ± 17	135 ± 24	136 ± 2	159 ± 1	164 ± 14	174 ± 6	179 ± 31	157 ± 5
#9	Left^a^	104 ± 7	***97*** ± ***19***	141 ± 12	158 ± 22	171 ± 16	193 ± 34	214 ± 15	185 ± 27	267 ± 16	227 ± 27
	Right	*57* ± *10*	75 ± 17	91 ± 5	108 ± 9	119 ± 4	138 ± 8	149 ± 2	128 ± 6	152 ± 6	181 ± 13
#10	Left^a^	79 ± 8	***66*** ± ***4***	106 ± 15	110 ± 18	112 ± 12	126 ± 7	129 ± 17	115 ± 4	123 ± 22	124 ± 15
	Right	65 ± 19	*58* ± *11*	97 ± 7	105 ± 9	116 ± 21	96 ± 15	102 ± 4	80 ± 8	100 ± 12	117 ± 7

Results for left and right foot are presented separately and data indicating the minimisation of peak pressure is in italics. The measurements for material BPU10 were used to identify the most heavily loaded foot (^a^) and to identify the overall optimum material (Bold) for each participant

As indicated in Table 3, in all cases the optimum BPU stiffness for quiet standing is found among the three softest materials, namely BPU01-03. The importance of correct selection was investigated by comparing the pressure reduction that was achieved by the optimum material against the respective pressure reductions achieved by the next softer and next stiffer one. One way repeated measures ANOVA with Bonferroni correction revealed that correct selection of insole stiffness has a significant effect on pressure reduction, Wilks’ Lambda = 0.196, F(2,7) = 14.32, *p* = 0.003. The value of multivariate partial eta squared was equal to 0.804 indicating a very large effect size. Indeed, across all participants the average (±STDEV) maximum pressure reduction achieved using the optimum BPU material was 42% (±15%). At the same time the average pressure reduction for the next softer and next stiffer material was 36% (±15%) and 30% (±12%) respectively, indicating an average improvement of 16 and 40% respectively. Pairwise comparison between the aforementioned conditions showed that the differences between optimum material and the next softer/next stiffer were statistically significant with *p* = 0.002/0.012 respectively.

Moreover Pearson correlation analysis revealed strong positive correlations between optimum relative stiffness (i.e., compressive stress for 50% strain) and the participants’ BM (*r* = 0.781, *N* = 10, *p* = 0.008) and BMI (*r* = 0.824, *N* = 10, *p* = 0.003). This finding indicates that stiffer materials could be needed in the case of participants with high BM or BMI, relatively to participants with lower BM or BMI, to minimise plantar pressure. No correlation was found between optimum relative stiffness for standing and the participants’ reference static peak pressure, height, or shoe size.

#### Walking

**Figure 4:**
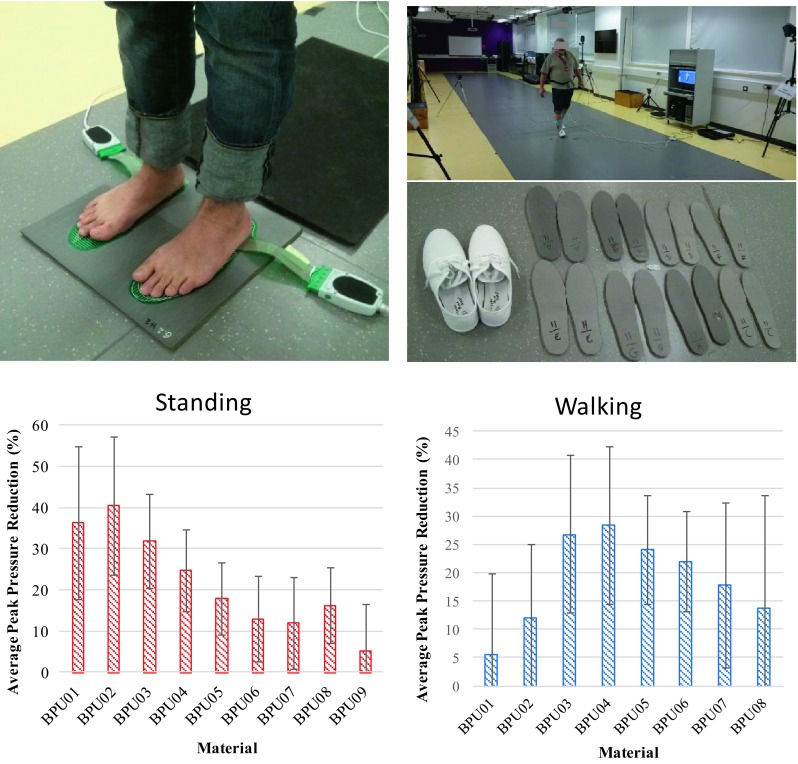
The testing set-up (Top) and average pressure that was achieved by each material (bottom) during standing (left) and walking (right). Pressure reduction is averaged over ten participants.

During walking tests average walking speed ranged between 1.2 and 1.6 m/s. Pressure measurements during walking showed that eight out of ten participants loaded their left foot more heavily compared to their right foot (Table 4). Focusing on the most heavily loaded foot indicates that stiffer materials are likely to be needed to minimise pressure in the case of walking compared to standing (Fig. 4). Indeed, optimum materials for walking range between material BPU03 and BPU06 (Table 4).

**Table 4:** Average (± STDEV) maximum peak pressure for walking (dynamic peak pressure) on different BPU materials for all participants.

Participant	Foot	Dynamic peak pressure (kPa)									
		BPU01	BPU02	BPU03	BPU04	BPU05	BPU06	BPU07	BPU08	BPU09	BPU10 (reference)
#1	Left^a^	420 ± 17	453 ± 13	***291*** ± ***6***	311 ± 26	453 ± 3	419 ± 40	–	–	–	550 ± 8
	Right	318 ± 19	368 ± 15	222 ± 25	*198* ± *17*	229 ± 30	282 ± 17	–	–	–	352 ± 5
#2	Left^a^	349 ± 17	348 ± 44	***293*** ± ***1***	327 ± 8	297 ± 14	333 ± 10	–	–	–	420 ± 2
	Right	306 ± 27	277 ± 19	236 ± 13	245 ± 21	*235* ± *5*	256 ± 6	–	–	–	302 ± 26
#3	Left^a^	429 ± 17	403 ± 4	***319*** ± ***8***	324 ± 13	331 ± 12	321 ± 5	–	–	–	507 ± 26
	Right	308 ± 26	323 ± 22	*263* ± *24*	266 ± 37	307 ± 13	288 ± 5	–	–	–	354 ± 51
#4	Left	774 ± 9	726 ± 47	*608* ± *53*	611 ± 48	620 ± 3	634 ± 37	664 ± 9	615 ± 32	–	719 ± 12
	Right^a^	871 ± 55	848 ± 52	675 ± 7	607 ± 44	587 ± 13	***585*** ± ***59***	588 ± 71	590 ± 32	–	818 ± 16
#5	Left^a^	445 ± 36	357 ± 88	333 ± 50	***283*** ± ***17***	388 ± 17	406 ± 8	–	–	–	566 ± 21
	Right	442 ± 6	402 ± 3	338 ± 29	*276* ± *5*	350 ± 85	314 ± 7	–	–	–	473 ± 20
#6	Left^a^	806 ± 20	746 ± 17	678 ± 21	641 ± 8	630 ± 10	***609*** ± ***4***	632 ± 6	685 ± 16	–	684 ± 11
	Right	690 ± 17	648 ± 24	522 ± 27	*457* ± *21*	460 ± 20	462 ± 6	494 ± 22	528 ± 26	–	550 ± 17
#7	Left^a^	465 ± 48	440 ± 30	381 ± 16	*343* ± *16*	360 ± 25	381 ± 9	–	–	–	461 ± 26
	Right	499 ± 11	495 ± 34	445 ± 11	***387*** ± ***6***	412 ± 6	417 ± 19	–	–	–	571 ± 15
#8	Left^a^	437 ± 4	417 ± 5	***383*** ± ***3***	384 ± 6	396 ± 1	394 ± 17	–	–	–	441 ± 25
	Right	370 ± 12	363 ± 5	339 ± 7	*333* ± *10*	338 ± 7	355 ± 4	–	–	–	437 ± 10
#9	Left^a^	581 ± 36	450 ± 38	***401*** ± ***10***	443 ± 41	428 ± 17	481 ± 20	–	–	–	540 ± 25
	Right	546 ± 28	502 ± 35	*361* ± *21*	375 ± 17	394 ± 31	415 ± 17	–	–	–	478 ± 23
#10	Left^a^	346 ± 58	320 ± 16	225 ± 16	***212*** ± ***42***	223 ± 52	261 ± 31	–	–	–	338 ± 63
	Right	341 ± 25	312 ± 9	239 ± 19	*206* ± *15*	274 ± 24	278 ± 8	–	–	–	320 ± 17

Results have been averaged over at least 12 mid-gait steps. Even though all BPU materials are presented in the table some of them were excluded from these tests (for being too stiff) and no results are presented for them. Results for left and right foot are shown separately and data indicating the minimisation of peak pressure is in italics. The measurements for material BPU10 were used to identify the most heavily loaded foot (^a^) and to identify the overall optimum material (bold) for each participant

One way repeated measures ANOVA with Bonferroni correction indicated that correct selection of cushioning material’s stiffness has a significant effect on pressure reduction during walking, Wilks’ Lambda = 0.283, *F*(2,8) = 10.15, *p* = 0.006. The value of multivariate partial eta squared was equal to 0.717 suggesting a very large effect size. Across all participants the average (±STDEV) maximum pressure reduction achieved using the optimum BPU material was 31% (±13%). At the same time the average pressure reduction for the next softer and next stiffer material was 21 (±11) and 26% (±11%), respectively, indicating an average improvement of 48 and 19% respectively. Pairwise comparison between the three conditions showed that the differences between optimum material and the next softer was statistically significant with *p* = 0.011, while the difference between optimum material and the next stiffer was borderline non-significant with *p* = 0.050.

Pearson correlation analysis revealed strong positive correlations between the optimum relative stiffness and the participants’ BM (*r* = 0.738, *N* = 10, *p* = 0.015) and BMI (*r* = 0.797, *N* = 10, *p* = 0.006) indicating that optimum insole stiffness increases with BM and BMI. Moreover strong positive correlations were also found between reference dynamic peak pressure and the participants’ BM (*r* = 0.799, *N* = 10, *p* = 0.006) and BMI (*r* = 0.894, *N* = 10, *p* < 0.001), indicating that pressure also increases with BM and BMI. No statistically significant correlation was found between optimum relative stiffness and the participants’ reference dynamic peak pressure, height, shoe size or walking speed.

## Discussion

Previous research has highlighted the importance of careful selection of cushioning materials’ stiffness to achieve maximum pressure reduction.^6^,^9^,^20^ However, the actual importance of optimised material stiffness and its potential to improve pressure reduction has never been demonstrated *in vivo*. Moreover, currently no set method exists to help healthcare professionals working in the area of diabetic foot care to decide the best material for individual patients.^12^

In order to address these questions, a series of mechanical and *in vivo* tests were performed using bespoke cushioning materials. These materials were produced using chemical compounds that are commercially available and standard manufacturing techniques commonly used within the footwear industry (Supplementary material S1). This approach enabled investigating a very wide range of stiffness with materials that qualitatively exhibit similar stress/strain behaviour (Fig. 1a). In contrast to the bespoke materials, the two commercial materials that were used as reference (i.e., Astroshock^®^ and Poron^®^4000) have similar relevant stiffness but their stress/strain behaviour is distinctively different (Fig. 1b).

Previous numerical investigations performed by authors of this study, showed that loading is very likely to significantly influence the optimum cushioning properties of insole materials.^8^ For this reason, the ability of different cushioning materials to uniformly distribute externally applied forces was initially assessed under well controlled loading conditions using a 3D printed heel model. Similar testing techniques using rigid lasts or compression plates have been used before in the broader area of footwear biomechanics^21^ but not to quantify the ability of cushioning materials to uniformly distribute plantar loading.

In the case of the present study the direct measurement of pressure between the surrogate heel and cushioning materials for different magnitudes of externally applied force enabled the calculation of the relationship between force and peak pressure. Comparing these results between materials with different stiffness, revealed that different materials offer optimum cushioning for different magnitudes of compressive plantar load. More specifically it is indicated that stiffer materials are needed in the case for higher loads in order to minimise pressure (Fig. 3).

One could argue that the use of a surrogate heel (even of an anatomically accurate one) cannot simulate the unique mechanical characteristics and loading of the human heel. Indeed the stiffness of the surrogate heel pad is very different compared to a real heel pad. However, besides its limitations this test offers new and valuable insight on the link between loading and optimum cushioning. Even though the absolute values of results cannot be translated to *in vivo* conditions, the comparative results for different materials can significantly enhance the interpretation of *in vivo* results by eliminating the variability that characterises *in vivo* testing. The validity of these comparative results is also supported by a previous numerical study where the optimum insole stiffness was found not to be affected by the stiffness of the heel pad.^8^

*In vivo* testing under static and dynamic conditions was performed to identify the BPU materials that achieve maximum pressure reduction during standing and walking. For the purpose of this study the material that achieved maximum pressure reduction for the most heavily loaded foot was considered to be the optimum one. This hypothesis was tested by comparing the pressure reduction that was achieved by the optimum material against materials with lower or higher stiffness. One way repeated measures ANOVA revealed substantial and statistically significant improvement in pressure reduction for the case of optimum material, as this was defined here. For example, in the case of walking, optimum material stiffness led to pressure reduction of 31% (±13%), but changing the optimum material to the next softer or next stiffer material reduced pressure reduction to 21% (±11%) or 26% (±11%) respectively. This analysis highlighted the importance of careful selection of cushioning materials’ and for the first time enabled quantifying *in vivo* the improvement optimised stiffness can achieve in terms of pressure reduction.

One of the most interesting findings of this study was the strong positive correlation between optimum material stiffness and the participants’ BM and BMI. These correlations indicate that people with higher BM or BMI might need stiffer insole/footwear materials in order to minimise plantar pressure. Considering the results of mechanical testing presented here as well as previous numerical results^8^ the observed increase in optimum stiffness with increasing BM or BMI can be attributed to increased loading magnitude. This is also supported by the strong positive correlations found between reference dynamic peak plantar pressure during walking and the participants’ BM and BMI.

From an engineer’s point of view the relationship between increased BM or BMI and increased in-shoe plantar pressure seems to be intuitively correct. This relationship is also supported by literature and strong evidence for people with no known musculoskeletal pathology or diabetes^2^,^18^ as well as for people with diabetes and neuropathy.^1^ However clear consensus has not been yet reached and further testing is needed.^7^

Even though more correlations could be explored, these findings indicate that optimum stiffness could possibly be predicted without the need for lengthy and expensive measurements such as gait analysis and plantar pressure measurements. Subject to further testing and validation, this can set the basis for simple methods to inform material selection and/or material optimisation in the clinic. The importance of BM for the selection of materials in foot orthoses has been previously highlighted but only based on qualitative observations.^15^

Within-subject comparisons also indicated that the materials that minimised pressure during standing were different to those that minimised pressure during walking, with stiffer materials needed in the latter case (Fig. 4). Indeed, assuming stress for 50% quasi-static compression describes the relative stiffness of cushioning materials during standing (static stiffness) and stress for 50% dynamic compression (Table 1) describes relative stiffness during walking (dynamic stiffness) it is calculated that in average the optimum dynamic stiffness is 337% (±149%) higher than the optimum static one. This substantial increase in optimum relative stiffness highlights also the need for optimised viscoelastic properties in order to achieve maximum pressure reduction both in highly dynamic (e.g., fast walking, running *etc*.) and less dynamic activities of daily living (e.g., standing, slow walking *etc*.). With regards to clinical practice, this finding indicates that different cushioning materials could be needed for people leading highly active lives compared to people with more sedentary lifestyles. Even though investigating optimum viscoelastic properties of cushioning materials was beyond the scope of this study, the results presented here set the basis for further analysis in this area.

Finally, the comparison between the results for left and right foot of each participant revealed that in many cases different materials may be needed to minimise pressure for each foot, which raises the question of optimising cushioning properties separately for left and right foot. Using different material to minimise pressure could further enhance the offloading capacity of therapeutic footwear and orthoses, assuming that it doesn’t have a detrimental effect on gait and postural balance. The observed difference between optimum stiffness for left and right can be explained by differences in loading, which in turn can be explained by asymmetry in gait. Indeed asymmetry during able-bodied ambulation is well established in literature and linked to functional differences between limbs.^19^,^22^

Although previous studies indicate that changing footwear sole stiffness has an effect on spatio-temporal parameters of gait in normal participants,^23^ because of the focus on diabetic neuropathy and the structure of the present study the aforementioned changes in gait were not assessed. This could be the subject to further investigations, however it could be anticipated that this effect would be less prominent in people with impaired sensation in their feet, namely people with diabetic neuropathy. This is evidenced in a recent study exploring static balance in people with diabetic neuropathy.^16^ Despite that, further testing involving people with diabetic foot is of paramount importance to verify the transferability of the findings of this study to clinical populations.

At this point it needs to be highlighted that all pressure measurements mentioned in this paper correspond to the entire area of the foot. Therefore, the findings about the effect of material stiffness on optimum cushioning properties are restricted to the reduction in the overall peak pressure. This simplification was deemed to be a necessary first step to shed light on the importance of correct material selection and material optimisation.

Specifically in the case of standing, participants were asked to stand as still as possible and try not to change the way they stand between conditions. However no actual control over the movement of their centre of pressure (COP) was in place to completely eliminate the possibility of COP moving between forefoot and rearfoot between conditions. Having said that, it should also be noted that the reliability of observations is enhanced by the fact that the same trends were found in standing and walking.

As a next step, detailed region-specific analyses are needed to develop clinically relevant strategies for pressure reduction. These strategies will have to take into account changes in pressure distribution between different phases of gait and the effect of foot morphology (e.g., flat foot vs. pes cavus *etc*.). Strategies for targeted pressure reduction would be particularly relevant in the case of people with foot deformities or any other pathology that affects loading.^5^

The thickness of the custom cushioning materials was close to the top end of the range that is used for the fabrication of insoles.^14^ However, the fact that the material’s stress/strain behaviour remains the same regardless of the sample’s thickness means that the pressure leading to bottoming out will also remain the same. Based on that it can be concluded that optimum stiffness is unlikely to be affected by the cushioning material’s thickness. At this point it should be highlighted that the findings of this study are relevant to any cushioning material used in footwear and not just to insoles. Indeed the findings about optimum cushioning indicate that the combined compressive stiffness of the entire foot-bed (i.e., combination of outer-sole, midsole and insole) could be optimised to achieve maximum pressure reduction.

For the first time this study provides strong quantitative data to support the importance of stiffness optimisation in cushioning materials in order to promote the even distribution of externally applied forces. This finding is particularly relevant in the case of therapeutic footwear where plantar pressure reduction is a key therapeutic objective. Finally, the results of this study highlight the need for more testing to support the development of clinically relevant methods for material selection.

## Electronic supplementary material

Below is the link to the electronic supplementary material.

Supplementary material 1 (PDF 689 kb)
