# Supplementary material for: Subject Specific Optimisation of the Stiffness of Footwear Material for Maximum Plantar Pressure Reduction
Source: Ann Biomed Eng. 2017 May 9;45(8):1929–40. doi: 10.1007/s10439-017-1826-4 (PMC5527058; doi:10.1007/s10439-017-1826-4)
Supplement: Supplementary file 1 — Supplementary material 1 (PDF 689 kb) [file 10439_2017_1826_MOESM1_ESM.pdf]

<u>Manufacturing of bespoke polyurethane foam materials</u>

Polyurethane (PU) foams are made from two liquid components:

(A) Polyether polyol, catalysts, surfactant, water and additives

(B) Isocyanate

Changing the relative ratio of components A and B in the formulation of the PU foam enables tailoring their mechanical characteristics. The specific formulations used in this study are presented in table 1. The manufacturer for the chemicals used to make the bespoke PU foams was BEIL GmbH (Moderne Orthopädie Kunststoffe, Lehmkuhlenweg 9, DE-31224 Peine, GERMANY).

<table>
<tr><th>Material name in manuscript</th><th>Composition commercial name</th><th>Component A (g)</th><th>Component B (g)</th><th>Total mixture mass (g)</th><th>Mould volume (L)</th></tr>
<tr><td>BPU01</td><td>Asti S 100/54</td><td>32.47</td><td>17.53</td><td rowspan="2">50</td><td rowspan="10">0.4</td></tr>
<tr><td>BPU02</td><td>Asti S 100/62</td><td>30.86</td><td>19.14</td></tr>
<tr><td>BPU03</td><td>AstiTech® 150 100/42</td><td>94.37</td><td>39.63</td><td rowspan="5">134</td></tr>
<tr><td>BPU04</td><td>AstiTech® 150 100/46</td><td>91.78</td><td>42.22</td></tr>
<tr><td>BPU05</td><td>AstiTech® 150 100/50</td><td>89.33</td><td>44.67</td></tr>
<tr><td>BPU06</td><td>AstiTech® 150 100/54</td><td>87.01</td><td>46.99</td></tr>
<tr><td>BPU07</td><td>AstiTech® 150 100/58</td><td>84.81</td><td>49.19</td></tr>
<tr><td>BPU08</td><td>AstiTech® 300 100/42</td><td>133.8</td><td>56.2</td><td rowspan="3">190</td></tr>
<tr><td>BPU09</td><td>AstiTech® 300 100/50</td><td>126.67</td><td>63.33</td></tr>
<tr><td>BPU10</td><td>AstiTech® 300 100/58</td><td>120.25</td><td>69.75</td></tr>
</table>

<u>Table 1:</u> The composition of the formulations that were used to produce bespoke PU foam materials BPU01-BPU10.

Both components are very sensitive to humidity and temperature variations which makes temperature control of the raw materials extremely important. During storage temperature of ingredients was kept between 21°C and 32°C.

According to typical moulding technique, a 200mm x 200mm x 10mm aluminium mould (Figure 1) was preheated to 60°C and coated with two different mould release agents: a solid mould release agent (*ACMOS P180-52*) followed by a liquid one which was sprayed (*ACMOS 37-5224*). A preheated mixture of components A and B (table 1) was then poured in the mould before sealing it to allow the formation of the PU foam (Figure 1).

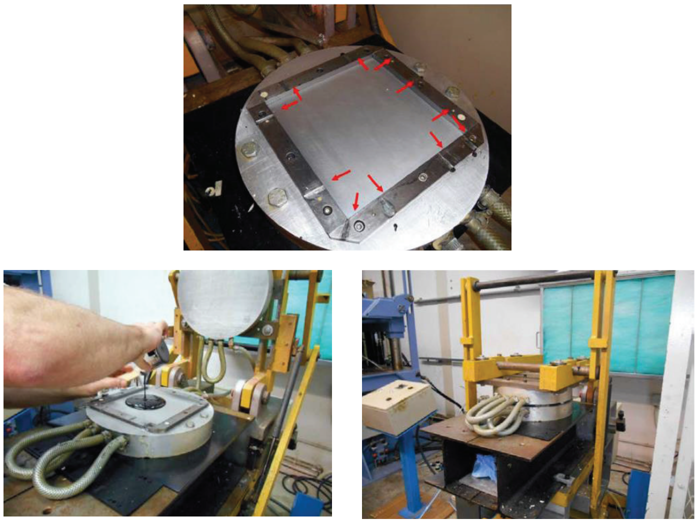

Figure 1: Overview of the moulding system.

The natural colour of PU foams is white. In this case colorant (1÷ 3%) was added to component A before mixing it with component B to colour the material sheets grey/ black (Figure 2).

In order to allow curing the PU foam sheets were left to relax for at least 24 hours at room temperature, prior to any testing.

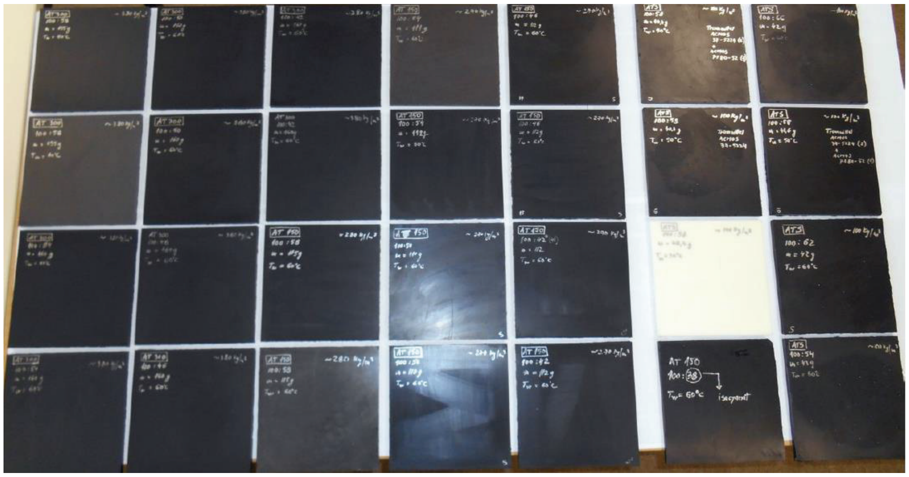

Figure 2: Typical moulded PU foam material sheets

.
